# Getting into host’s skin: initial immune response to *Schistosoma mansoni* infection

**DOI:** 10.3389/fimmu.2025.1661465

**Published:** 2025-12-17

**Authors:** Carolina Melo Orrico-Ferreira, Paulo Emilio Oliveira Cruz, Joao Vitor Borges Rios, Wellington da Silva Rosa, Beatriz de Souza Santos, Raphael Chagas Silva, Carina Silva Pinheiro, Barbara Castro-Pimentel Figueiredo

**Affiliations:** 1 Serviço de Imunologia, Hospital Universitário Prof. Edgar Santos, Universidade Federal da Bahia, Salvador, Brazil; 2 Programa de Pós-graduação em Imunologia, Universidade Federal da Bahia, Salvador, Brazil; 3 Laboratório de Alergia e Acarologia, Universidade Federal da Bahia, Salvador, Brazil; 4 Departamento de Bioquímica e Biofísica, Universidade Federal da Bahia, Salvador, Brazil

**Keywords:** cercaria, cercarial elastase, immune response, schistosome infection, skin immunity

## Abstract

*Schistosoma mansoni* initiates infection through active skin penetration by cercariae, triggering early immune responses that are crucial to disease establishment. In naïve hosts, parasite antigens elicit an initial Th1-type response that is rapidly modulated to a Th2 profile by parasite-derived immunomodulatory molecules, including proteases and eicosanoids. Upon reinfection, prior sensitization enhances Th2 responses, IgE production, and eosinophil recruitment, while promoting regulatory mechanisms that limit pathology and favor chronic infection. Key molecules such as cercarial elastase (SmCE) interfere with antigen presentation and antibody function, facilitating immune evasion. This review explores the early immune responses elicited at the cutaneous interface during primary *S. mansoni* infection and reinfection, emphasizing the dynamic interplay between host defense mechanisms and parasite-driven immunoregulation. Understanding these early skin-stage events reveals how *S. mansoni* balances immune activation and suppression, offering valuable targets for vaccine development and immune-based interventions.

## 1 Introduction

Schistosomiasis is a parasitic disease caused by trematodes of the genus *Schistosoma* and is classified among the neglected tropical diseases (NTDs). This disease is frequently associated to increasing burdens on public health systems, especially in developing countries. Schistosomiasis is characterized by significant heterogeneity in infection patterns and disproportionately affects rural populations lacking adequate sanitation. Depending on local habits and socioeconomic conditions, such as the use of contaminated water for agriculture or recreation, clinical cases can vary considerably even within a single community (1). According to the latest WHO report, *Schistosoma mansoni* remains a major public health concern, with approximately 251.4 million people worldwide requiring preventive chemotherapy in 2021. The *S. mansoni* is endemic in 55 countries, including many in Africa, as well as parts of South America (notably Brazil, Venezuela, and Suriname) and the Caribbean, highlighting the ongoing need for targeted control efforts and expanded treatment access (2). Among the *Schistosoma* species infecting humans, *Schistosoma mansoni*, *Schistosoma haematobium* and *Schistosoma japonicum* are the most prevalent, while *Schistosoma guineensis*, *Schistosoma intercalatum* and *Schistosoma mekongi* have restricted prevalence in specific regions of the world (3). *S. mansoni* and *S. haematobium* are endemic in Sub-saharan Africa, whereas *S. mansoni* is the only endemic species in South America and especially in Brazil. In Asia, *S. japonicum* is the most common, followed by *S. mekongi* (2, 3). According to the Global Burden of Disease Study 2021, schistosomiasis cases reported in that year were predominantly concentrated in Africa, South America, and China. Globally, an estimated 151.377 million people were affected by schistosomiasis in 2021, with the regions classified as having the lowest socio-demographic Index (SDI) exhibiting the greatest disease burden The highest number of cases occurred in Western sub-Saharan Africa (61.535 million), followed by Eastern sub-Saharan Africa (46.949 million) and East Asia (11.460 million) (4, 5).

The *Schistosoma mansoni* life cycle is complex and requires two distinct hosts to be completed: a freshwater snail of the genus *Biomphalaria* as the intermediate host, and humans as the definitive host (3, 6). Human schistosomiasis begins when individuals come into contact with freshwater sources containing *S. mansoni* cercariae, the free-swimming larval stage released by infected snails. These infective larvae actively penetrate human skin and, once inside the host, transform into schistosomula and migrate through the dermis into the vasculature, eventually reaching the mesenteric veins, where they mature into adult worms. There, the adult worms reproduce sexually and release numerous eggs, some of which become trapped in host tissues, whereas others are excreted in the feces, continuing the cycle upon reaching freshwater. In the aquatic environment, miracidia hatch from the deposited eggs and infect *Biomphalaria* snails, where they undergo asexual reproduction and generate new cercariae (7). The *S. mansoni* life cycle therefore relies on precise coordination between environmental conditions, invertebrate and vertebrate hosts, and complex developmental transitions.

When *S. mansoni* cercariae actively penetrate human skin, they trigger early inflammatory responses that initiate the immunomodulatory processes necessary for infection establishment and parasite development (7). Penetration begins when the motile cercaria contacts the host’s skin, propelled by vigorous tail movements. Additional structures, such as the cercarial head and acetabular glands, play key roles in this process, releasing immunologically active molecules that engage the host immune system (8). Although this phase represents a critical step in disease establishment, it remains incompletely understood. Primary immune responses arise within hours of infection, with notable differences observed between naïve and previously exposed individuals. While these early events are decisive for the success or failure of infection, the molecular triggers and cellular dynamics involved remain poorly characterized. Thus, advancing the understanding of the parasite’s early developmental stages, its secreted metabolites, and their immunological functions is essential for elucidating the mechanisms of host invasion and improving strategies for interrupting transmission. Although much is known about the immunopathology associated with egg deposition and chronic disease, the very first moments of cercarial skin invasion represent a crucial yet underexplored phase of host-parasite interaction. In this review, we examine current knowledge on the immune mechanisms and molecular mediators involved in the host’s initial response to *S. mansoni* cercarial infection, highlighting some parasite-derived proteins that contribute to immunomodulation and immune evasion at this early stage. Although this is not a systematic review, the literature selection process followed the main principles of the PRISMA 2020 reporting guideline to ensure transparency, objectivity, and reproducibility (9). Detailed methodology, a PRISMA flow diagram and the completed PRISMA checklist are provided as supplementary file (**Supplementary figure 01**).

## 2 Parasite life cycle and infection through skin penetration

The complex life cycle of *S. mansoni* alternates between free-living aquatic stages and parasitic stages within intermediate (snails) and definitive hosts (mammals). Infected mammals release parasite eggs into freshwater bodies, such as rivers and lakes, via fecal excretion. Under favorable environmental conditions, such as hypotonicity, adequate temperature, and light exposure, miracidia hatches from the eggs and actively infects the intermediate host: freshwater snails. Within the snail, the parasite undergoes asexual development, producing sporocysts that eventually generate cercariae, the mammalian-infective larval form. Once morphological and physiological mature, cercariae are released back into the water (10, 11). Upon skin contact with a suitable mammalian host, cercariae initiate the infection process. They respond to a combination of environmental factors, such as temperature and water viscosity (12) and host-derived signals, including surface lipids and L-arginine (13, 14) which collectively trigger their activation and guide their orientation toward the skin surface.

Cercariae use vigorous tail movements to attach their heads, via their oral and ventral suckers, to the host epidermis, which is rich in keratinocytes and Langerhans cells (LC). During this process, the parasite secretes proteolytic enzymes from its acetabular glands, primarily serine proteases, secreted either via extracellular vesicles or as soluble excretory/secretory (E/S) products, to degrade key structural proteins of the stratum corneum and underlying epidermal layers thereby enabling entry (15). Importantly, these molecules not only disrupt epithelial barriers but also constitute the first schistosome-derived antigens encountered by host immune cells, thereby contributing to the initiation of early immune responses (7). The cercaria then sheds its tail and undergoes morphological transformation into a schistosomulum, adapting to survival within host tissues. As the parasite migrates through the skin, it promotes alterations to the tegument and upregulation of immune-evasion proteins. This active and enzyme-mediated invasion strategy allows schistosomes to bypass both physical and immune barriers at the skin and initiate systemic infection.

Once inside the circulation, schistosomula travel to the lungs, and subsequently to the hepatic portal system, where they mature into adult worms (10, 11). Sexual reproduction occurs in the mesenteric vasculature, and the eggs produced can traverse the intestinal epithelium to be excreted in feces, thus completing the life cycle upon contact with freshwater (11). Early interactions between invading cercariae and the skin’s structural and immunological components are critical in shaping the host’s initial immune response, setting the stage for downstream immunomodulatory events that define schistosomiasis pathogenesis. In this review, particular emphasis is placed on the molecular and cellular mechanisms occurring in the skin, highlighting both host defense and parasite-driven immunomodulation.

## 3 Initial immune response to schistosome infection

Upon cercarial penetration of the definitive host’s skin, *S. mansoni* infection is initiated. Experiments using murine keratinocytes stimulated with cercarial E/S antigens demonstrated that these cells are able to produce innate pro-inflammatory mediators IL-1α and IL-1β, supporting their role as precursors in initiating cutaneous schistosome immune responses (16, 17). Cytokines released by keratinocytes contribute to the recruitment and activation of dermal immune cells, establishing the initial inflammatory environment that links innate and adaptive responses. This early activation precedes the systemic Th1-type response observed during the acute phase of infection.

In the course of schistosomiasis, the Th1 response is modulated by the production of interleukin-10 (IL-10) and later shifts toward an antigen-driven Th2-type response (18, 19). IL-10 plays a key immunoregulatory role by inhibiting the production of pro-inflammatory mediators such as interferon-gamma (IFN-γ), tumor necrosis factor-alpha (TNF-α), and nitric oxide (NO), thereby contributing to immune homeostasis (18). Experiments conducted in murine models demonstrate that IL-10-mediated functions include T cell suppression, inhibition of dendritic cell (DC) differentiation, modulation of macrophage activation, and regulation of both Th1 and Th2 cytokine profiles (20). IL-10 production by CD4^+^ T cells in the skin results from both an early response directed against skin-resident commensal bacteria and the emergence of schistosome-specific CD4^+^ T cell responses over subsequent days (21). Together, these immune mechanisms contribute to the control of inflammation and minimize tissue damage associated with *Schistosoma* infection.

The early immune response triggered during cercarial invasion and schistosomulum migration through the skin remains considerably less studied than the well-characterized immune responses to adult worms and to granulomas formed around parasite eggs. Nevertheless, it is known that several factors - whether parasite-derived or host-related - can influence the magnitude and nature of this initial immune response. A critical factor influencing the immune-response profile is the parasite sex, as host immune response can differ depending on whether infection occurs with male or female parasites. In single-sex infection murine models, male and female schistosomes have opposing immunomodulatory effects: females suppress early immune responses and promote anergy, whereas males enhance innate immunity, reducing parasite burden (22). Regarding the host, the immune response varies depending on whether the host is naïve or has had prior exposure to *S. mansoni* antigens from the infective stage. In the following sections, we outline the current understanding of the different immune response profiles associated with the infectious process.

### 3.1 Primary immune events following first-time *S. mansoni* infection

During the first exposure to *S. mansoni*, naïve hosts respond to cercarial invasion and the early stages of schistosomulum migration through the activation of innate immune mechanisms triggered by parasite antigens. As reviewed by McMannus and colleagues (2018), during percutaneous penetration by cercariae, few larvae die within the skin, whereas the majority enter the venous circulation through small blood or lymphatic vessels (3, 7). In naïve individuals, the initial contact with parasite antigens, released from acetabular glands and present on the larval surface, initiates a robust immune response involving multiple host cells and signaling molecules. This infection may elicit a significant inflammatory response in the skin, often manifested as a transient pruritic maculopapular reaction known as cercarial dermatitis (23). Such cutaneous immune reaction may precede the systemic manifestations of acute schistosomiasis, commonly referred to as Katayama fever, particularly in previously unexposed individuals who acquire schistosomiasis after traveling to endemic areas (23, 24). In contrast, individuals living in endemic regions for schistosomiasis rarely present cercarial dermatitis upon first *S. mansoni* infection because of pre-existing immune resistance, frequently resulting from prenatal exposure to *S. mansoni* antigens and/or transplacental transfer of anti-helminthic antibodies (25). Nonetheless, cercarial dermatitis may still occur in cases of repeated reinfection with *Schistosoma mansoni*.

Experimental studies have provided mechanistic insight into these cutaneous manifestations. Studies employing human skin organ culture demonstrated that, demonstrated that *S. mansoni* schistosomula remain predominantly within the epidermis during the first 24 hours after penetration, reaching the dermis only after 48 hours and approaching dermal vessels by approximately 72 hours post-infection. Cytokine analysis at early time points revealed increased expression of IL-1Ra, IL-10, and TNF-α, indicating that the initial host response to cercarial invasion involves both pro-inflammatory and regulatory mediators that likely contribute to the controlled inflammatory profile observed during early infection (26).

In murine models, exposure to radiation-attenuated schistosome larvae revealed key tissue and immune responses that occur shortly after parasite entry. Upon cercarial penetration, a series of immediate immune and tissue responses are triggered. Early studies using mice exposed to cercariae describe epidermal thickening, edema, dilation of peripheral blood vessels, and focal inflammatory infiltrates in the dermis (27). Experiments in mice using radiation-attenuated (RA) schistosome larvae have also shown the influx of immune cells into the skin, leading to increased tissue thickness, driven by the secretion of chemokines and cytokines (28). These early infiltrates are rich in neutrophils, which actively phagocytose secretions from acetabular glands (7, 27–29). Paveley and colleagues (2009) demonstrated, using fluorescent cercaria in murine infection experiments, that within three hours post-infection, neutrophils become the predominant cells at the site of infection, efficiently engulfing fluorescently labeled cercarial secretions. Neutrophils were also found adhered to the parasite’s membrane *in vitro*, particularly in the presence of active complement, although only a small fraction of these cells (approximately 5%) release their granules onto the parasite surface (30). Cercarial proteins stimulate neutrophils to produce chemokines that recruit other immune cells, including monocytes, macrophages, and DCs (29, 30). Although eosinophils are rarely observed at this early stage in the first *S. mansoni* infection, they may contribute to parasite damage through membrane stripping mediated by C3/C3-receptor interactions (31). *In vitro* studies demonstrated that eosinophils from normal rats can adhere to and kill schistosomula within 18 hours through complement activation via the alternative pathway, independently of antibodies, suggesting that complement-mediated eosinophil adherence represents an important mechanism of parasite destruction in the host (31). Together, these leukocytes promote the release of inflammatory cytokines and create an IL-4 and IL-13-rich environment in schistosome-exposed mice (29).

In parallel with cellular responses, the complement system also becomes rapidly activated in the skin following cercarial invasion. As newly transformed schistosomula reach the dermis, the complement system is activated via the alternative pathway, contributing to larval death (32). *In vitro* studies have shown that schistosomula incubated with immune serum from schistosomiasis patients are efficiently killed, whereas incubation with fetal calf serum does not affect parasite viability (32). Further analyses revealed that exposure of the parasite surface to human serum triggers deposition of the membrane attack complex (MAC) on the tegument, resulting in parasite killing, although a significant proportion of schistosomula (up to 30%) can still survive after six days in culture (33). Early in their maturation, schistosomula develop resistance to complement-mediated killing through the rapid and extensive release of acetabular proteases and shedding of the glycocalyx coat (34, 35). Beyond its direct lytic activity, complement activation also enhances the immune response by promoting the release of chemoattractants for neutrophils and eosinophils and facilitating their adherence to the parasite. Within hours, complement may also induce mast-cell degranulation, leading to histamine release and increased vascular permeability (7, 35). Nevertheless, experiments using C3-deficient rat serum have shown that complement plays a more prominent role during the late phase of larval migration (8–13 days post-infection), with a limited contribution to the early innate response against radiation-attenuated cercariae (36).

In addition to complement activation, innate immune cells rapidly accumulate at the site of cercarial entry. Within three hours of cercarial invasion, macrophages (CD11b^+^/F4/80^+^, MHC class II^+^) are recruited to the site of radiation-attenuated cercariae entry in mice skin (7). Innate immune cells surrounding the parasite phagocytose acetabular gland components (29), and secrete pro-inflammatory molecules such as MIP-1α, MIP-1β, IL-6, and IL-1β (7). Although macrophages can eliminate schistosomula independently of complement or antibodies, their adherence is enhanced by IgE immune complexes (30).

**Figure 1:**
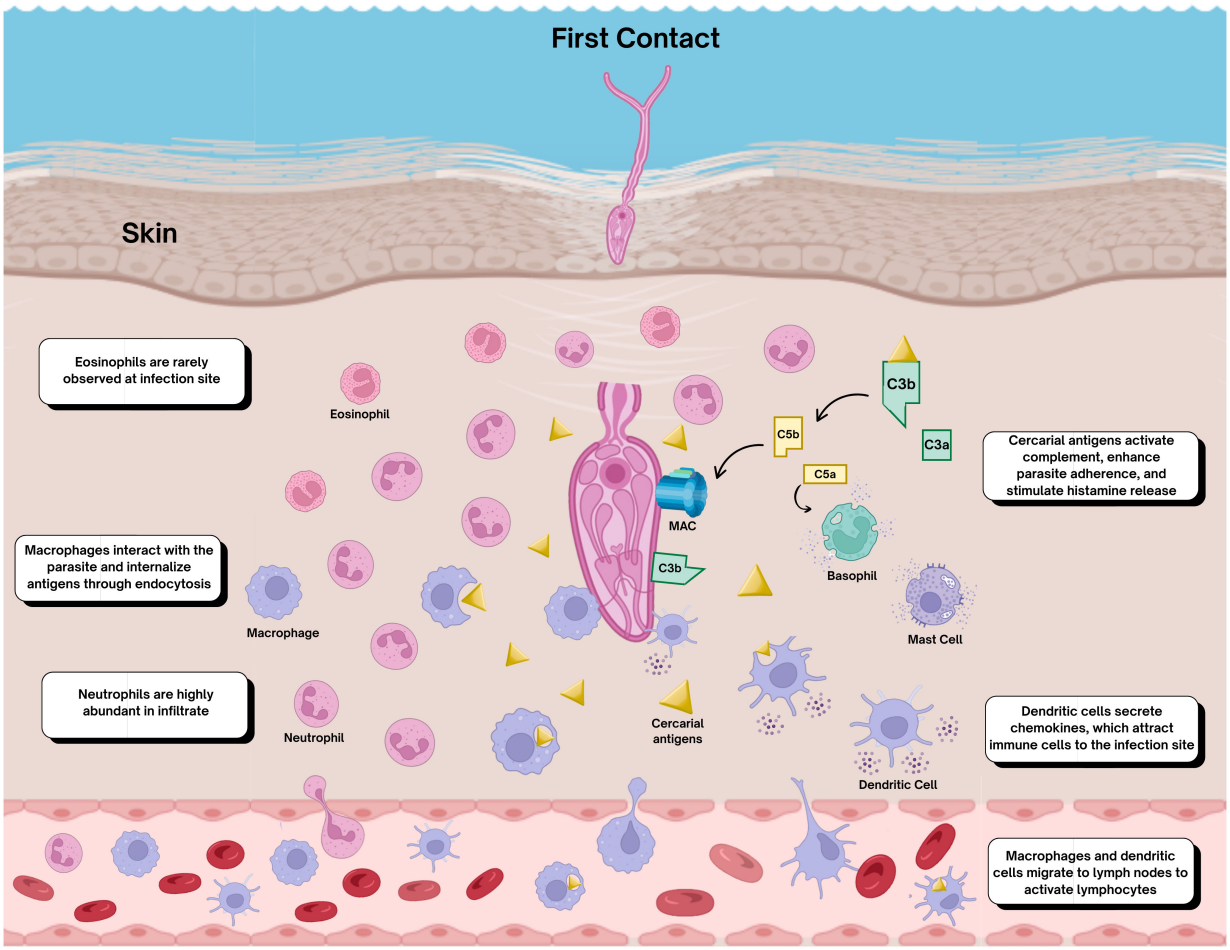
Early immune responses in the skin following primary *Schistosoma mansoni* cercarial exposure. Upon first contact, cercarial penetration activates epidermal keratinocytes and resident sentinel cells, inducing a localized pro-inflammatory response characterized by neutrophil recruitment and classical macrophage activation. At this stage, eosinophils are scarce, and immune activation is dominated by innate mechanisms. Figure created with BioRender.com.

At this stage of infection, neutrophils dominate the cellular infiltrate (**Figure 1**). As the response progresses, macrophages and DCs become central in orchestrating the cutaneous immune response to invading schistosomes. Infection of mice with fluorescent cercariae demonstrated that E/S products released by schistosomula in the first three hours post-transformation contain proteases and immunomodulatory molecules and activate macrophages via MyD88-dependent pathways, suggesting involvement of Toll-like receptors. These products also stimulate DCs that promote strong Th2 responses and secrete higher levels of IL-6, TNF-α, IL-12p40/23, and iNOS (29). Phenotypic analysis of dermal DCs from infected human skin explants revealed expression of regulatory markers PD-L1 and PD-L2 and production of IL-10 (37). After antigen uptake, both DCs and macrophages migrate to draining lymph nodes, where they present *S. mansoni* antigens to naïve T cells via MHC class II^+^ cells.

This inflammatory scenario begins to shift around two to four days post-infection, as most larvae enter the bloodstream (7). The circulatory system transports the larvae (now termed schistosomula) to the liver, where they will continue their maturation. By this time, the skin microenvironment contains elevated levels of both Th1- and Th2-type cytokines, and CD4^+^ T cells become activated and migrate to the lungs, where they play a key role in host protection. Meanwhile, the draining lymph nodes of infected mice represent a Th1-polarized cytokine environment (7). At this point, lymphocyte maturation and IgE production begin, priming the immune system for distinct responses upon subsequent *S. mansoni* exposures. A summary of the main immune cells involved and their respective roles during primary infection is provided in **Table 1**.

**Table 1:** Immune cells involved in the response to *Schistosoma mansoni* cercarial infection.

Cell type	First-time *S. mansoni* infection	*S. mansoni* reinfection	References
Neutrophils	- Highly abundant in early infiltrates (2–3 h post-penetration). - Phagocytose acetabular gland secretions. - Produce chemokines that recruit monocytes, macrophages, and DCs. - Complement-driven recruitment.	- Reduced frequency in infiltrate. - Adhere to parasite membrane, less firmly than eosinophils. - Still involved in phagocytosing secretions.	(7, 44)
Eosinophils	- Rare during early response - May contribute to parasite damage via complement-mediated membrane stripping - Secrete IL-4 and IL-13.	- Predominant granulocyte at infection site (~2.5 h). - Adhere to parasite via Fc–Fc interaction with IgG. - Contribute to a Th2 cytokine secretion (IL-4, IL-13).	(7, 29, 44)
Mast Cells	- Secrete histamine and vasoactive amines following activation by parasite antigens or complement. - Contribute to early vascular changes.	- Activated by antigen–IgE interaction. - Release histamine and cytokines, facilitating eosinophil recruitment and tissue permeability.	(7, 53)
Macrophages	- Recruited to inflammation sit. - CD11b^+^/F4/80^+^/MHC II^+^ cells. - Phagocytose acetabular gland products. - MyD88-dependent cytokine secretion (e.g., IL-6, IL-1β) - Support TLR signaling.	- Functionally similar. - Produce IL-12 and IL-18 (~48 h post-infection). - Participate in immune regulation and tolerance via alternative activation.	(7, 29, 53)
Dendritic Cells	- CD11c^+^/MHC II^+^ cells. - Rapidly phagocytose parasite antigens - Initiate Th2 switching. - Secrete IL-6, TNF-α, IL-12p40/23, and iNOS. - Slower IL-10 production compared to macrophages.	- Similar profile - May have increased tolerogenic function. - Suppress Th1 polarization and promote IL-10 production in T cells. - Express CD86 and regulatory markers.	(7, 29, 53)
T Lymphocytes	- Not predominant in early stages. - Activated later via antigen presentation by DCs and macrophages in draining lymph nodes.	- CD3^+^/CD4^+^ T cells exhibit increased MHC II expression and reduced responsiveness - IL-10 and low Fas/FasL expression favor CD4^+^ T cell viability and hyporesponsiveness.	(7, 39, 40)

### 3.2 Immune events following *S. mansoni* reinfection

**Figure 2:**
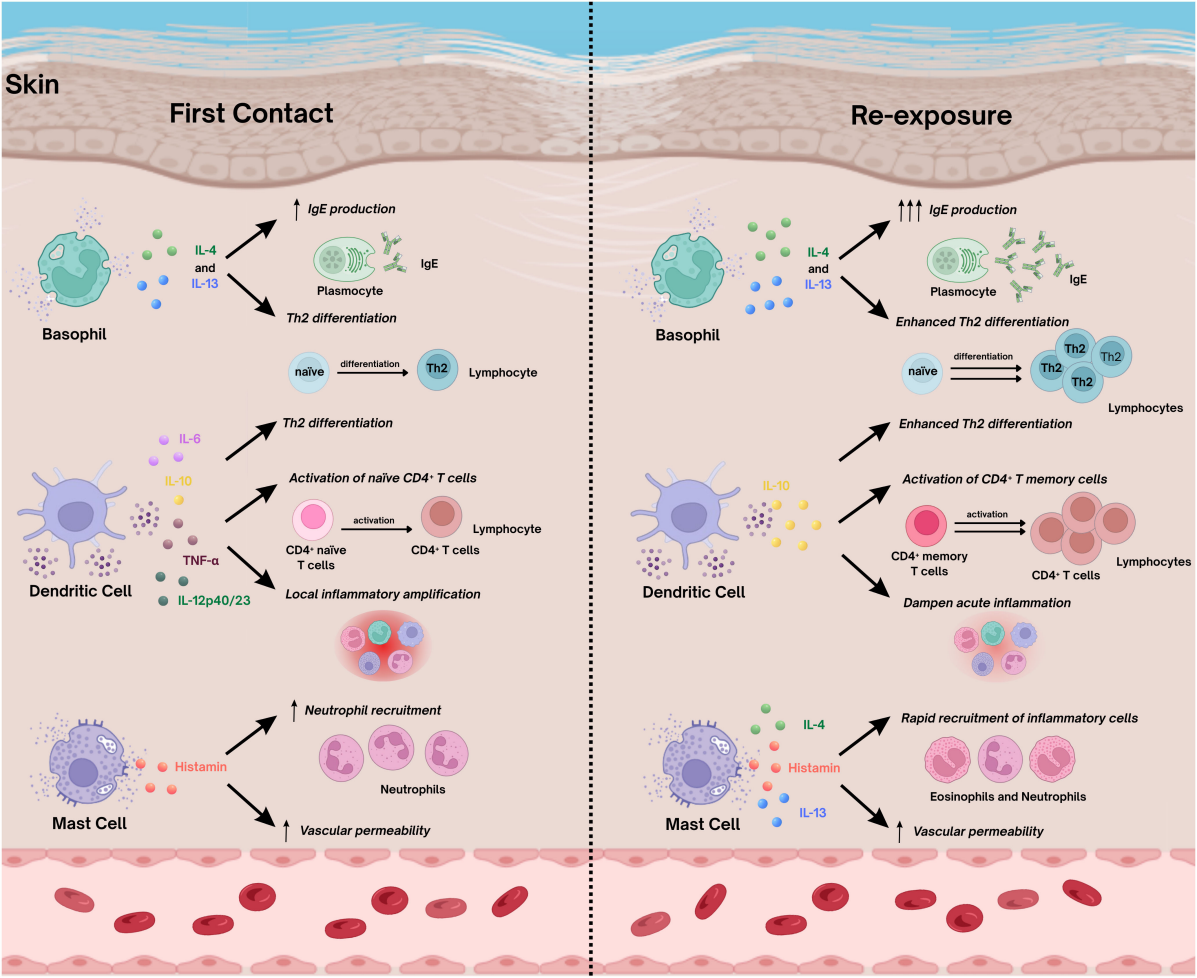
Cytokine production and functional interactions of immune cells during first contact and re-exposure to *Schistosoma mansoni*. Major mediators released by basophils, dendritic cells, and mast cells and their effects on local immune dynamics during first contact (left) and re-exposure (right). During first contact (left), basophils secrete IL-4 and IL-13 to initiate Th2 polarization and promote IgE synthesis. Dendritic cells release IL-6, IL-10, TNF-α, and IL-12p40/23, driving activation of naïve CD4^+^ T cells and amplifying local inflammation. Mast-cell degranulation results in histamine release, increased vascular permeability, and neutrophil recruitment. Upon re-exposure (right), enhanced type 2 cytokine secretion and elevated IgE production promote rapid activation of memory CD4^+^ T cells and accelerated recruitment of eosinophils and neutrophils. Together, these pathways shape the distinct immunological profiles observed during initial infection and reinfection. Figure created with BioRender.com.

While primary infection with *S. mansoni* elicits a robust innate response followed by Th1 and Th2 polarization, subsequent exposures are shaped by immunological memory established during prior infections. As cercariae penetrates the skin during reinfection, innate immune components, particularly keratinocytes and Langerhans cells (LCs), once again act as the first sensors of parasite entry, recognizing pathogen-associated molecular patterns (PAMPs), triggering a localized inflammatory response. Upon reinfection, hosts exhibit distinct immune profile characterized by accelerated cytokine production, enhanced effector cell recruitment, and increased regulation to limit tissue damage. Histological studies in murine models further illustrate these differences. In CBA mice, repeated exposure accelerates epidermal and dermal thickening and promotes a rapid shift from neutrophilic to eosinophilic infiltration at the penetration site, accompanied by microabscess formation and early parasite destruction (27). A comparative overview of cellular responses is presented in **Table 1** and **Figure 2**.

In murine models, repeated exposure to *S. mansoni* induces a shift toward the alternative activation of macrophage and reduced lymphoid responsiveness, altering cytokine production and modulating immune cell function. Notably, this favors the development of hyporesponsive T cells in an IL-4R-dependent manner (38). Moreover, repeated cercarial contact progressively conditions the host’s immune system toward tolerance, since IL-10-producing CD4^+^ T cells act locally to suppress excessive inflammation in the skin, thereby modulating both local and systemic responses to reinfection (21). The development of immune tolerance may benefit both host and parasite. In mice lacking the *RELMα* gene, a greater number of CD4^+^ T cells is found in the skin; however, these cells display reduced responsiveness (39). In murine models of repeated *S. mansoni* infection, cumulative percutaneous exposures result in the development of CD4^+^ T cell hyporesponsiveness. Mice lacking IL-4Rα exhibit with lower IL-10 levels in the skin, accompanied by increased numbers of activated MHC II^+^ antigen-presenting cells and CD4^+^ T cells. When associated with decreased expression of Fas/FasL, this leads to enhanced viability of CD4^+^ T cells (40).

Simultaneously, non-cytotoxic lymphocytes are present, releasing lymphokines that further stimulate the recruitment of more eosinophils and other mononuclear cells. Dendritic cells (DCs) and macrophages, which interact closely with T lymphocytes, are also regulated by the newly established immune environment. These cells perform similar functions similar to those observed in naïve individuals but are now closely associated with the polarization and presence of Th1 and Th2 subsets, as well as CD86^+^ and CD4^+^ T cells at the site of inflammation (7). Ex vivo analyses have shown that DCs suppress Th1 polarization of naïve T cells while increasing IL-10 production by T cells (37). Moreover, mice inoculated with irradiated cercariae display increased frequencies of CD206^+^ DCs and MHC II^+^CD86^+^ cells, indicating that these and other processes are dependent on parasite-secreted molecules (7).

**Figure 3:**
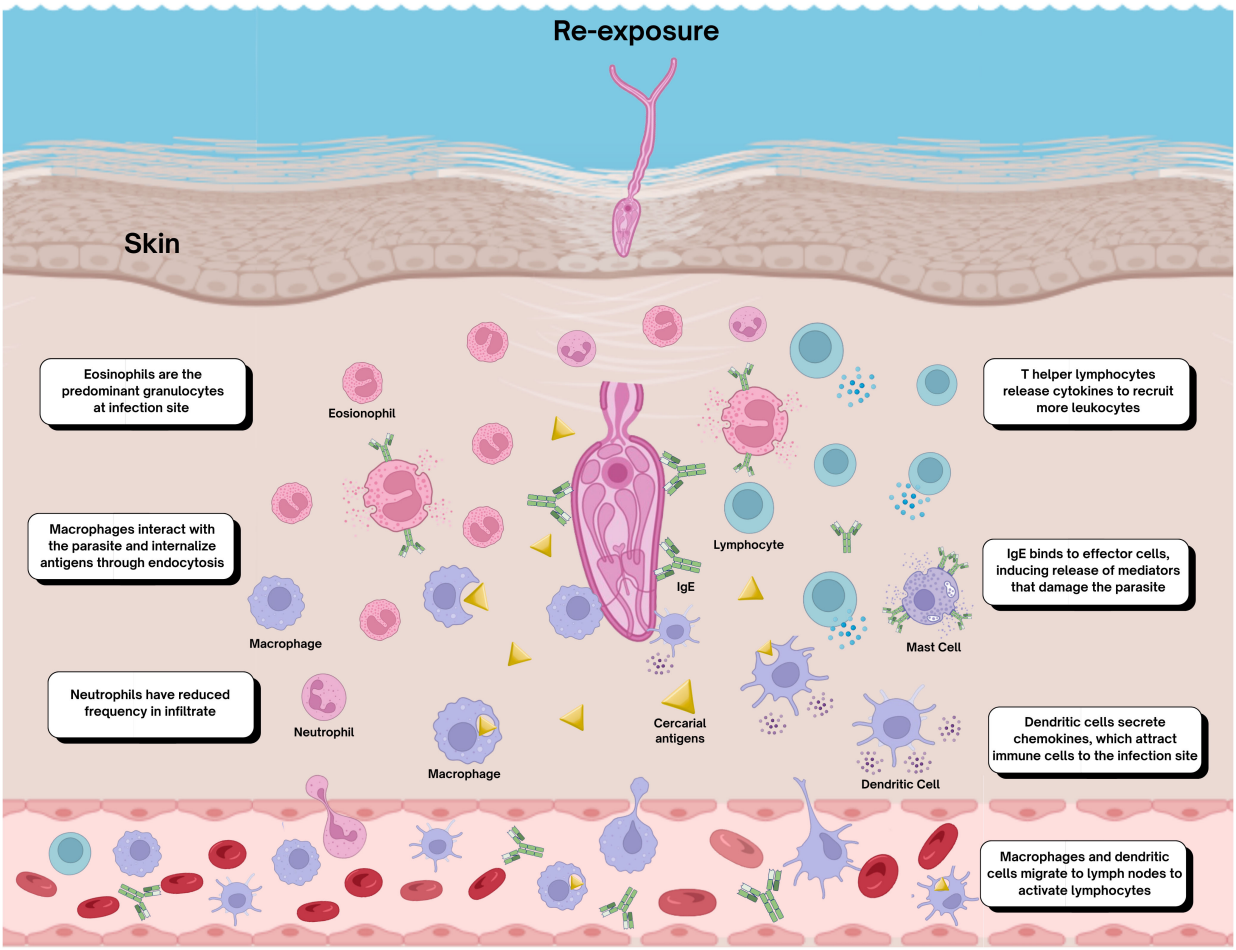
Early immune responses in the skin following reinfection with *Schistosoma mansoni*. Re-exposure to *Schistosoma mansoni* in previously infected hosts elicits a distinct immune profile, characterized by increased eosinophil infiltration, enhanced local IgE production, and activation of Th2 lymphocytes. Alternative macrophage activation predominates, accompanied by regulatory cytokines such as IL-10, which modulate local inflammation and contribute to immune tolerance. These events illustrate how prior antigenic exposure reshapes early cutaneous immune dynamics, influencing both parasite survival and systemic immune regulation. Figure created with BioRender.com.

In the early stages of schistosome reinfection, immunoglobulin E (IgE) plays a crucial role in orchestrating the immune response. IgE recognizes cercarial antigens and stimulates mast cells to release histamine and other vasoactive amines involved in angiogenesis and increased vascular permeability. These mediators, in combination with cytokines, contribute to the recruitment of immune cells such as eosinophils (41). Since eosinophil recruitment requires specific IgE, these cells are typically rare in the cellular infiltrates of naïve individuals. In contrast, eosinophils become the most abundant immune cells in the skin of previously exposed hosts, surpassing neutrophils - which dominate the early response in primary infections (42). This shift toward an eosinophil-rich infiltrate and increased IgE-mediated responses upon reinfection is graphically illustrated in **Figure 3**. Experimental studies in rats have shown elevated IgE levels in previously sensitized animals (43). The higher eosinophils counts in exposed individuals enhance the immune response, as these cells adhere to the schistosomula surface and induce larval damage, particularly through interactions with parasite-bound IgG (7). Eosinophil migration is stimulated by complement proteins, which enhance both activation and adherence to schistosomula. In murine models, eosinophil accumulation is driven by cytokines such as IL-13, which promotes recruitment to the infection site, and IL-4, which enhances IgE production. IgE subsequently binds to the parasite’s surface, forming immune complexes that also contribute to macrophage activation (34, 35, 44).

Finally, murine models demonstrate that reinfected hosts show accelerated granuloma formation and modulation, processes driven by pre-existing antibody and memory T-cell responses (41). These findings highlight the immunological complexity and dynamic regulation that characterize schistosomiasis reinfection, beginning at the site of cercarial penetration in the skin.

Studies discussed here were conducted primarily in murine models, with some comparative analyses in human skin explants. To clarify the experimental context and improve interpretability, **Table 2** summarizes the main studies that investigated early cutaneous immune responses to *S. mansoni* cercarial exposure, indicating the species or strain used, the anatomical site examined, and the time post-infection analyzed.

**Table 2:** Experimental models used to study early cutaneous immune responses to *Schistosoma mansoni* cercarial exposure.

Paper	Species/model	Skin context	Time window	Notes
Lichtenberg 1976 (42)	CBA and C57BL/6 mice	Dermal responses	6 - 48h	
Incani 1984 (27)	CBA mice	Epidermal and dermal responses	3 - 72h	
Fusco 1993 (49)	Living Skin Equivalent (LSE)	–	18 - 20h	
He 2002 (26)	Human skin organ cultures	Epidermal and dermal responses	2 - 72h	
Hogg 2003 (28)	C57BL/6 mice	Dermal responses	24 – 96h	Infection with radiation-attenuated cercaria
Kumkate 2007 (48)	C57BL/6 mice	Epidermal responses	0 - 96h	Infection with normal and radiation-attenuated cercaria
Paveley 2009 (29)	C57BL/6 mice	Epidermal and dermal responses	0 - 3h	Infection with fluorescent cercaria
Cook 2011 (38)	C57BL/6 mice	Epidermal and dermal responses	0h - 8 days	1× vs 4× cercarial exposure
Bourke 2015 (17)	C57BL/6 mice; keratinocytes	Epidermal responses	6 - 96h	
Prendergast 2015 (40)	C57BL/6 mice	Dermal responses	96h	Eosinophils were depleted from mice
Sanin 2015 (21)	C57BL/6 mice	Dermal responses	24 - 96h	Repeated cercaria exposure
Prendergast 2016 (39)	C57BL/6 mice	Dermal responses	96h	IL-4Rα KO and Relmα KO mice on C57BL/6 background
Sombetzki 2018 (22)	NMRI mice	Dermal responses	6 h	Cercariae were injected into air pouches in previously infected mice
Winkel 2018 (37)	human skin explants	Dermal responses	0,5 - 72h	Infection with normal and radiation-attenuated cercaria

## 4 Immunoregulation and immune evasion mechanisms during *S. mansoni* skin penetration

The events triggered during reinfection highlight not only immune adaptation but also the parasite’s capacity to modulate host defenses at the site of entry. While the host mounts memory responses, *S. mansoni* simultaneously initiates evasion strategies that interfere with immune recognition from the moment it breaches the skin, which acts as the first immunological barrier, capable of detecting and responding to *S. mansoni* cercariae. During penetration, *S. mansoni* cercariae must transverse the epidermis, where they can remain for at least 48 hours before reaching the dermis. From there, they rapidly gain access to blood and lymphatic vessels, allowing the parasite to progress through its life cycle. For this sequence of events to be successful, the parasite must exert control over the host’s immune responses.

Upon penetration, cercariae interact with epidermal keratinocytes and immune sentinel cells, triggering a localized pro-inflammatory response characterized by the release of chemokines and cytokines, such as IL-12 and IFN-γ, which drive Th1 polarization and macrophage activation (28). However, this early immune activation is rapidly modulated by the parasite, which promotes a shift toward a Th2 profile characterized by IL-4 and IL-10 production. IL-4 enhances IgE synthesis, while IL-10 dampens acute inflammation, facilitating parasite survival and immune evasion (45). This Th2 polarization benefits both the host, by limiting tissue damage, and the parasite, by enabling chronic infection. Notably, while this Th2 dominance in mice coincides with the onset of oviposition and granuloma formation, in humans and other models such as rats, the dynamics and timing of Th2 induction differ, reflecting host-specific immune regulation (40, 45).

In murine models, Langerhans cells (LCs) were found to be retained in the skin for up to 48 hours after cercarial exposure, which prevents their migration to draining lymph nodes and compromises antigen presentation to T cells (46). This inhibition is mediated by cercarial-derived lipid products, notably prostaglandin D_2_ (PGD_2_), which directly targets LCs and impairs their immunostimulatory function. Eicosanoids play a crucial role in modulating the immune response at the site of parasite entry, particularly by suppressing DC activity and promoting anti-inflammatory signaling. Among them, PGD_2_ secreted by the parasite impairs LC migration in an IL-10-independent manner (47). In experiments using radiation-attenuated larvae, migrating CD207^+^ LCs were detected in the dermis and skin-draining lymph nodes within 24–96 hours after exposure. However, LCs represented only a minor fraction of the total antigen-presenting cell population. Their limited numbers and similar migration kinetics in protective and non-protective infections suggest that LCs play only a marginal role in priming CD4^+^ T cells during early schistosomiasis (48). Together with PGD_2_, prostaglandin E_2_ (PGE_2_), produced by both host and parasite, further contributes to this immunosuppressive environment by inducing IL-10 secretion from alternatively activated macrophages and inhibiting DC maturation (49, 50).

Parasite-mediated immunoregulation involves adaptive mechanisms that enable cercariae to withstand the host’s immune defenses and establish infection (51). Through the release of a variety of E/S products with immunomodulatory activity (27, 46, 52), cercariae modulate local inflammatory signaling, promoting antibody cleavage, mast cell degranulation, complement activation, and cytokine production. In both murine skin and human keratinocyte models, cercarial secretions induce the production of the regulatory cytokine IL-10, which suppresses pro-inflammatory mediators and reduces the expression of MHC class II^+^ molecules, thereby limiting antigen presentation, T-cell activation, and IgE synthesis (28, 50, 52). Another key regulatory molecule identified at the penetration site is the interleukin-1 receptor antagonist (IL-1Ra), an inhibitor of IL-1–driven inflammation. IL-1Ra is produced by human keratinocytes in response to cercarial secretions, and this effect has been attributed to the E/S protein Sm16.8, identified in schistosomula secretions. Sm16.8 contributes to the anti-inflammatory environment in the skin by inducing IL-1Ra and suppressing antigen-induced lymphoproliferative responses, thereby facilitating parasite persistence and immune evasion (53).

Beyond cytokine modulation, *S. mansoni* employs a range of proteolytic enzymes and surface molecules that act directly on host tissues and immune components, further reinforcing its capacity for immune evasion during skin invasion. Cercariae and schistosomula also release surface molecules and enzymes, derived from the glycocalyx and acetabular gland secretions, that directly suppress immune cells activation and help maintain a balance between Th1 and Th2 immune responses. *In vitro* studies have shown that early larval E/S products inhibit peripheral blood mononuclear cell activity (47). Within the first three initial hours post cercariae transformation into schistosomula, these products induce mast cell degranulation and histamine release independent of IgE, subsequently stimulating IL-4 and IL-10 production, which dampen Th1 responses (47). Among these secreted molecules, particular attention has been given to specific proteases with dual roles in tissue invasion and immune modulation.

The cercarial invadolysin SmCI-1, a metalloprotease secreted from the acetabular glands, contributes to immune evasion by cleaving host proteins such as collagen type IV, fibrinogen, and complement component C3b. This cleavage inhibits both the classical and alternative complement pathways, protecting schistosomula from complement-mediated lysis. SmCI-1 also promotes an anti-inflammatory microenvironment by enhancing IL-10 production and suppressing pro-inflammatory cytokines and eosinophil-associated mediators. These effects collectively facilitate parasite survival during early skin invasion (54).

The cercarial elastase of *S. mansoni* (SmCE) stands out as the most abundant and functionally significant enzyme involved in parasite penetration (16). The sequencing of the *S. mansoni* genome revealed a gene family encoding isoforms of the cercarial elastase enzyme (SmCE), comprising eight full-length genes. Based on sequence identity, these isoforms were grouped into two major classes: Group I (SmCE 1a, 1b, 1c) and Group II (SmCE 2a, 2b). Protein expression has been confirmed for SmCE 1a, 1b, and 2a, while SmCE 1c was classified as a pseudogene, and no protein product has been detected for SmCE 2b transcripts (55, 56). Despite sequence divergence, the SmCE isoforms display similar expression profiles throughout the parasite’s life cycle, with SmCE 1a being the most abundantly expressed. Bioinformatic analyses suggest that the isoforms share comparable substrate specificities, supporting the hypothesis that SmCE variants are functionally redundant (55). Proteomic studies have further confirmed the significance of SmCE in parasite invasion. Cercarial extracts containing SmCE have been shown to cleave several human proteins, and functional assays have validated the enzyme’s ability to degrade at least seven different dermal proteins (57), underscoring its critical role in active skin penetration and early immune evasion by the parasite. SmCE is implicated in immune evasion by lysing dermal proteins, degrading IgE, and possibly stimulating IL-10 production by macrophages (51, 58, 59).

**Figure 4:**
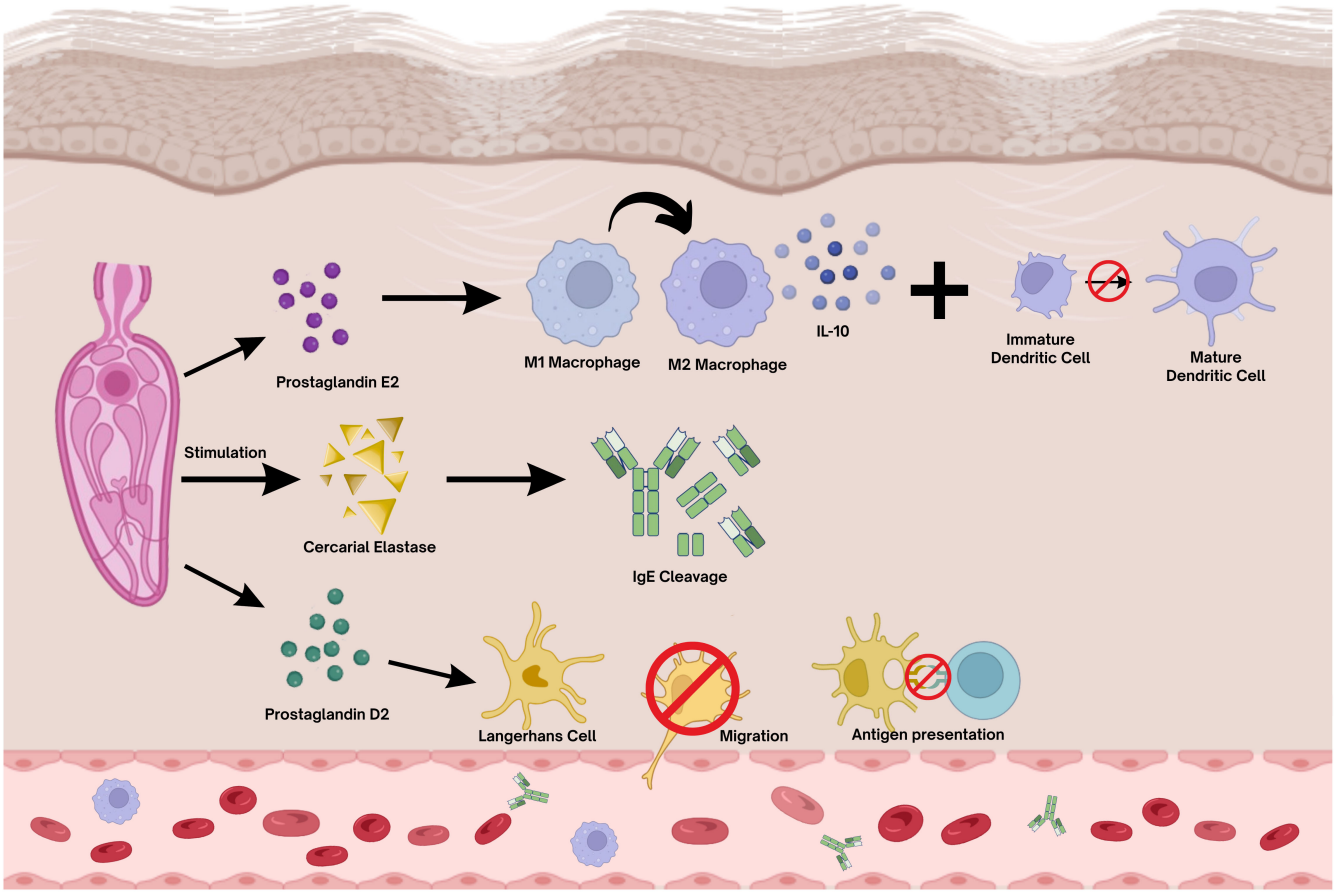
Immunomodulatory effects of *Schistosoma mansoni* cercaria. During skin penetration, cercaria release a variety of E/S products with potent immunomodulatory properties that alter local inflammatory signaling. Among these, cercarial-derived lipid mediators, notably prostaglandin D_2_ (PGD_2_), impair Langerhans cell (LC) migration to draining lymph nodes, thereby impairing antigen presentation to T lymphocytes. In parallel, prostaglandin E_2_ (PGE_2_), produced by both host and parasite, reinforces this immunosuppressive environment by inducing IL-10 secretion from alternatively activated macrophages and inhibiting DC maturation. In addition, the cercarial protease elastase contributes to IgE cleavage, reducing antigen-specific recognition and further hindering T-cell activation. Together, these mechanisms promote immune evasion at the skin interface, facilitating parasite survival and successful establishment of infection. Figure created with BioRender.com.

In reinfected individuals, antibodies play a central role in recognizing and neutralizing cercarial antigens. However, *S. mansoni* cercariae possess proteolytic mechanisms capable of cleaving specific immunoglobulins before they can effectively bind to parasitic epitopes. Proteases secreted during skin penetration not only degrade extracellular matrix components to facilitate migration but also target host antibodies, particularly IgE (60). Cercarial and early schistosomula extracts can cleave IgE from humans, rats, and mice, while leaving IgA and IgG largely unaffected (58, 59). To identify the specific enzymes responsible, researchers tested purified cercarial elastase and confirmed its capacity to cleave the Fc region of IgE, effectively inactivating the antibody (51). Notably, the cleavage site targeted by cercarial elastase is conserved across several immunoregulatory proteins, including FcγRI, IL-2, IL-10R, IL-12R, and TLR3. These findings suggest a dual role for cercarial elastase in both dermal invasion and immune evasion, as it disables key components of the host’s early immune response. Continued investigation into cercarial proteases may reveal promising vaccine targets or therapeutic strategies aimed at preserving antibody function and enhancing protective immunity against reinfection. Altogether, these findings highlight the multifaceted strategies employed by *S. mansoni* to modulate host immunity from the moment of skin penetration, as summarized in **Figure 4**.

## 5 Summary and prospects

In conclusion, the occurrence and intensity of *S. mansoni* transmission are determined by environmental factors that affect the survival and abundance of intermediate host snails and the viability of free-living larval stages, including water temperature, pH, turbidity, vegetation and rainfall patterns, as well as anthropogenic factors such as sanitation infrastructure and water-contact behaviors. These determinants vary substantially across regions and influence exposure intensity and seasonal transmission dynamics. Such ecological variation can modulate population-level infection risk but does not, by itself, constitute evidence of intrinsic differences in host cutaneous immune mechanisms among human populations.

Beyond understanding environmental conditions that determine *S. mansoni* infection, elucidating the early immune events that unfold during skin penetration is critical to unraveling the complex host-parasite interactions that define disease establishment and determine infection outcome. This review highlights the dynamic and multifaceted immune responses triggered upon *S. mansoni* cercarial penetration, emphasizing how innate and adaptive mechanisms vary significantly between primary and repeated exposures. The initial innate responses are dominated by Th1 cytokines, neutrophils, and antigen-presenting cells, and are rapidly modulated by parasite-derived E/S products. Molecules such as cercarial elastase (SmCE), prostaglandins, and IL-1Ra shift the response toward a Th2 profile marked by IL-4 and IL-10, thereby promoting parasite survival and priming adaptive immunity. In reinfected hosts, prior sensitization leads to a qualitatively distinct immune profile characterized by faster Th2 activation, IgE-driven eosinophilia, and regulatory cytokines, resulting in more effective larval control but also tolerance-inducing mechanisms that favor chronic infection.

Taken together, the findings discussed in this review underline skin as a central immunological interface in schistosomiasis, where the balance between resistance and tolerance is orchestrated. Yet, despite growing insights into the early events following cercarial entry, many questions remain. The parasite’s ability to manipulate both innate and adaptive responses underscores the need for identifying novel immunomodulatory molecules involved in immune evasion. Moreover, current evidence is insufficient to determine whether the early cutaneous immune response to *S. mansoni* differs systematically across human populations. A deeper understanding of this early cutaneous immune response will likely shape future research efforts and highlight several promising targets for vaccines and immune-based therapies aimed at enhancing protective responses, mitigating schistosomiasis morbidity, and limiting transmission.
