# Supplementary material for: Getting into host’s skin: initial immune response to Schistosoma mansoni infection
Source: Front Immunol. 2025 Dec 17;16:1661465. doi: 10.3389/fimmu.2025.1661465 (PMC12753927; doi:10.3389/fimmu.2025.1661465)
Supplement: Supplementary file 1 [file DataSheet1.pdf]

Supplementary methodology

Although this is not a systematic review, the literature selection process followed the main principles of the PRISMA 2020 reporting guideline to ensure transparency, objectivity, and reproducibility (1). All searches were conducted using the PubMed online platform (https://pubmed.ncbi.nlm.nih.gov/). The search terms included the following keywords: *"cercaria"*, *"immunity"*, *"penetration"*, and *"Schistosoma mansoni"*. The retrieved publications were screened qualitatively, and those meeting the inclusion criteria were considered for the bibliographic review.

We considered the following inclusion criteria: (i) articles providing information on immunological events triggered during the penetration of *S. mansoni* cercariae; (ii) studies reporting immune responses associated with proteins secreted by cercariae or schistosomula; and (iii) studies describing immune evasion mechanisms of cercariae or schistosomula. Moreover, exclusion criteria were: (i) manuscripts written in languages other than English, Portuguese or Spanish; (ii) documents not classified as original scientific articles (e.g., editorials, notes, or reviews); and (iii) unavailability of the full text in the consulted databases.

Based on these criteria, the literature search and selection process proceeded through four stages: identification, screening, eligibility, and inclusion. Initially, 57 articles were identified using the selected keywords in PubMed. During the screening phase, all retrieved records were assessed against the inclusion and exclusion criteria. In the eligibility phase, 25 articles were excluded for the following reasons: 15 studies were out of scope, 6 lacked accessible full-text versions, and 4 were written in languages other than English, Portuguese or Spanish.

As a result, 32 articles were included in the qualitative synthesis. Each selected paper was then critically assessed not only for its scientific content but also for indicators of reliability, including journal impact factors, number of citations, and the journal's historical reputation within the field. In addition, new manuscripts cited in the reference lists of the selected articles were examined, and those meeting all inclusion criteria were incorporated into the review.

The workflow followed for article identification, screening, and inclusion is illustrated in **Supplementary Figure 1**, which depicts the sequential steps adapted from the PRISMA flow diagram.

(1) Page MJ, McKenzie JE, Bossuyt PM, Boutron I, Hoffmann TC, Mulrow CD, et al.
The PRISMA 2020 statement: an updated guideline for reporting systematic reviews. *BMJ* 2021;372:n71. doi:10.1136/bmj.n71

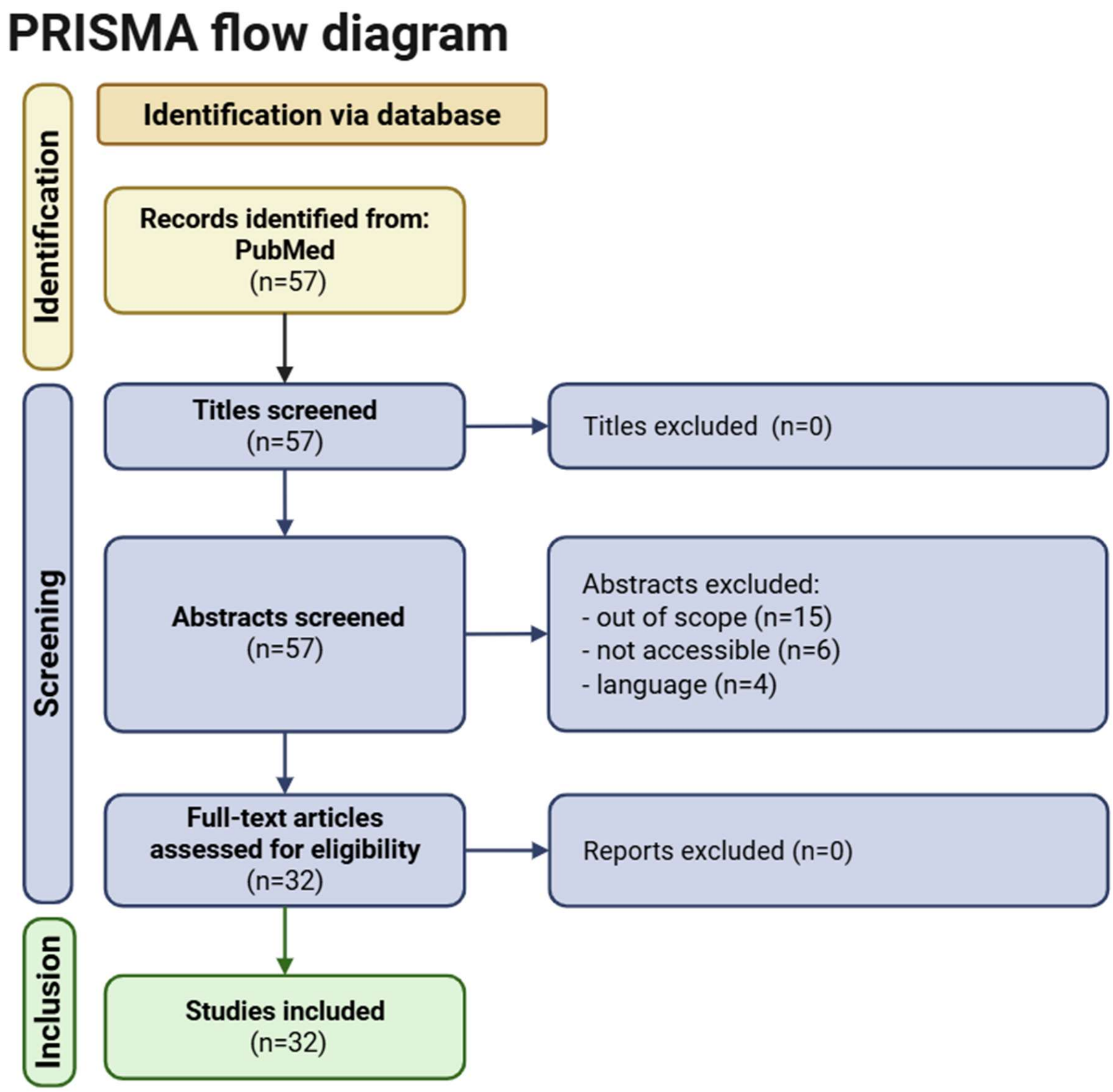

Sup. Figure 1 - Flow diagram illustrating the article selection process used in this review. During the identification stage, 57 articles were retrieved from the PubMed database using the selected keywords. These publications were evaluated during the screening phase, and those meeting the inclusion criteria were assessed for eligibility. A total of 25 articles were excluded after full-text evaluation for the following reasons: 15 were out of scope, 6 did not provide access to the complete manuscript, and 4 were written in languages other than English. Consequently, 32 articles were included in the qualitative synthesis that formed the basis of this review. Figure created with BioRender.com.
